# The Design, Synthesis, and Characterization of Epoxy Vitrimers with Enhanced Glass Transition Temperatures

**DOI:** 10.3390/polym15224346

**Published:** 2023-11-07

**Authors:** Chunai Dai, Yang Shi, Zhen Li, Tingting Hu, Xiao Wang, Yi Ding, Luting Yan, Yaohua Liang, Yingze Cao, Pengfei Wang

**Affiliations:** 1 School of Physical Science and Engineering, Beijing Jiaotong University, Beijing 100044, China; chadai@bjtu.edu.cn (C.D.); 19126255@bjtu.edu.cn (Y.S.); 23126690@bjtu.edu.cn (X.W.); 23121824@bjtu.edu.cn (Y.D.); ltyan@bjtu.edu.cn (L.Y.); 2 China Academy of Aerospace Science and Innovation, Beijing 100088, China; 3 Department of Agricultural and Biosystems Engineering, South Dakota State University, Brookings, SD 57007, USA; 4 China Academy of Space Technology, Beijing 100094, China

**Keywords:** epoxy vitrimer, glass transition temperature, epoxy resin E51, curing agent, phthalic anhydride, sebacic acid

## Abstract

A series of epoxy vitrimers (EVs) with enhanced glass transition temperatures (*T*_g_s) were synthesized by curing epoxy resin E51 with different ratios of phthalic anhydride and sebacic acid as curing agents, and 1,5,7-triazabicyclic [4.4.0] dece-5-ene as a transesterification catalyst, and their curing dynamics, rheological properties, mechanical properties, and thermal stability were comprehensively investigated. By adjusting the molar ratio of the anhydride to the carboxylic acid in the curing agent, the *T*_g_s of the EVs increased from 79 to 143 °C with the increase in the anhydride content. In particular, the material EV-5.5 with a high usable *T*_g_ of 98 °C could undergo stress relaxation through the transesterification reaction when exposed to high temperatures (160 to 200 °C), and the correlation between the relaxation time and temperature follows the Arrhenius equation. Moreover, EV-5.5 exhibited elastomeric behavior, where brittle fractures occurred before yielding, which demonstrated a tensile strength of 52 MPa. EV-5.5 also exhibited good thermal stability with a decomposition temperature (*T*_d5_) of 322 °C. This study introduces new possibilities for practical applications of thermoset epoxy resins under special environmental conditions.

## 1. Introduction

Epoxy resins are frequently used as coatings, flame retardant materials, structural materials, and semiconductor component packaging materials in the application fields of construction, electronics, automotives, aerospace, and so on, due to their excellent mechanical properties, good heat resistance and chemical resistance, and good adhesion to a variety of substrates [1,2]. However, since epoxy resins are thermosets with internal polymer chain cross-linked structures that are three-dimensional and permanent, making them insoluble and infusible, it is difficult for them to be recycled and reused once they have been shaped, which limits their applications to a large extent and results in a waste of resources [3].

In 2011, Leibler and coworkers [4] used zinc acetate and zinc acetyl acetonate as catalysts to catalyze the classic epoxy chemistry reaction of the diglycidyl ether of bisphenol A and fatty dicarboxylic and tricarboxylic acids, and obtained a cure containing a large number of dynamic exchange ester bonds. Thanks to the transesterification reaction (TER), the classic thermoset epoxy resin was fluid while maintaining the integrity and insolubility of its cross-linked network at high temperatures and could be reshaped and reprocessed. Such polymers with covalently cross-linked networks and glass-like fluid properties are therefore called vitrimers [5]. After Leibler’s group proposed the TER-based epoxy vitrimer (EV), numerous researchers developed vitrimers based on disulfide bonds [6,7,8,9,10], amino exchange [11,12], olefin metathesis [13,14] and dynamic imine bonds [15,16,17], which greatly enriched the vitrimer system. However, due to the fact that the introduction of dynamic bonds mostly requires special functional groups, and the preparation processes are complex or costly, the research on EVs continues to focus on the creation of dynamic covalent bonds via TER [18,19,20].

Most TER-based EV materials are prepared on the basis of acid anhydride- or carboxylic acid-cured epoxy resins. The raw materials for epoxy resins are diverse and so are the curing agents, and the key to the preparation of EVs is the addition of the appropriate catalysts to initiate the TER and catalyze curing reactions. The present catalysts mainly include organic salts (zinc acetate, zinc acetyl acetonate, dibutyltin diacetate, etc.), strong bases (1,5,7-triazide bicyclic (4.4.0) dece-5-ene (TBD), etc.) [20,21] and so on. Currently, most researchers in this field are focusing on the effects of bio-based resin feedstocks [22,23], curing agent types, catalyst types or catalyst-free conditions, epoxy group functionality, and material grain size on the cross-linking process and the mechanical properties of EV materials [21], which have facilitated the development of diverse applications, such as chemical recycling and self-healing, energy storage, electronic devices, shape-shifting materials and devices, artificial muscles, and microfabrication [24,25,26,27,28,29].

The ultimate potential of vitrimers, however, is limited by the ability to tune the glass transition temperature (*T*_g_) for the target application. At elevated temperatures, vitrimers exhibit the characteristics of viscoelastic fluids, displaying flow behavior. Conversely, at lower temperatures, the exchange reactions within vitrimers occur at a significantly slower rate, giving them characteristics resembling those of conventional thermoset materials. The transition from a liquid-like state to a solid state is reversible and corresponds to a glass transition phenomenon [30]. The *T*_g_ is the critical parameter that demarcates the transition between a glassy state and a rubbery state, which is of significant relevance in determining the temperature at which vitrimers become activated [31]. Advanced and smart EV materials have sparked our interest in their application to aerospace vehicles. Aerospace vehicles are a special target application case. Because the exposed components of aerospace vehicles frequently reach temperatures beyond 90 °C when operating continuously under the special environment conditions of air resistance and solar radiation [32], the suitable EV materials are suggested to have an appropriate *T*_g_ of above 90 °C while maintaining certain mechanical properties. For this purpose, we concentrate on the design, preparation, and characterization of EVs with enhanced *T*_g_s exceeding 90 °C.

*T*_g_ is the temperature at which the chain segments in the polymer change from the frozen state to the moving state, and the movement of the chain segments is realized through the internal rotation of single bonds on the main chain. Hence, the flexibility of the polymer chain has an influence on the *T*_g_ of the polymer. Binary and ternary fatty acids tend to have long flexible chains connected via multiple single bonds, so the *T*_g_s of epoxy resins or vitrimers cured with them are typically low, mostly less than 50 °C [30]. Currently, researchers primarily employ two methods to produce epoxy resins or vitrimers with elevated *T*_g_s. The first approach involves the use of epoxy monomers and curing agents that incorporate rigid groups, such as phenyl and biphenyl, or multifunctional groups. This strategy aims to enhance the rigidity of the molecular chains and the cross-linking density of the cured products, thereby augmenting the resistance to molecular chain segment movement [33,34,35,36]. For example, Wu and coworkers [37] used such an effective strategy, where natural glycyrrhizic acid (GL) with sebacic acid (SA) were used as curing agents to prepare TER-based EVs. The prepared V4 exhibited a fast stress relaxation (a relaxation time of 130 s at 180 °C) and a usable *T*_g_ of 61 °C. An alternative approach is to combine epoxy with other resins with high *T*_g_s, such as benzocaine and cyanate. Through copolymerization, the resulting product could exhibit a *T*_g_ exceeding 150 °C [38,39]. However, improving the *T*_g_, network rearrangement rate, and mechanical properties simultaneously to meet material requirements in the special environment of aerospace vehicles remains a big challenge for TER-based EVs [40].

Based on previous reports, our group investigated the use of some curing agents containing rigid groups in the original epoxy–carboxylic acid vitrimer system. The preliminary experiments revealed that aromatic carboxylic acids such as isophthalic acid and terephthalic acid demonstrated higher melting points (greater than 200 °C) and lower reaction temperatures with the epoxy group (the reaction could occur at 130 °C under the condition of TBD catalyst), which resulted in poor process performance; 4,4′-diaminodiphenylmethane with a melting point of 90 °C could be used to cure the diglycidyl ether of bisphenol A or E51 to enhance the *T*_g_ of the products, but high-*T*_g_ systems suffered from insufficient TERs due to the lack of ester bonds and the low network mobility.

**Figure 1:**
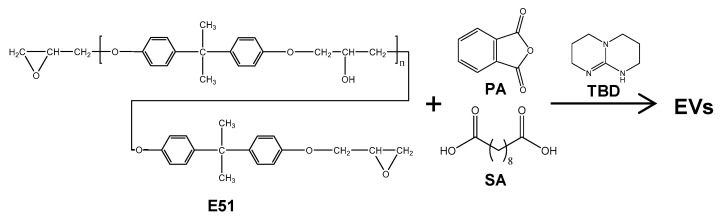
Curing reaction of EVs synthesized from E51, PA, and SA using TBD as the catalyst.

Finally, in this study, a determined curing agent containing rigid groups employed to cure epoxy resin oligomers is reported. Specifically, epoxy resin E51 was utilized as the monomer, while a mixture of phthalic anhydride (PA) and sebacic acid (SA) in varying proportions served as the curing agent. The curing reaction took place in the presence of the transesterification catalyst of TBD. As a result, EVs with elevated *T*_g_s were successfully synthesized (Figure 1), which introduce new possibilities for practical applications of thermosets such as healing or convenient processability in a wider temperature range. Additionally, the created EV-5.5 with a *T*_g_ of 98 °C had a suitable stress relaxation rate as well as strong mechanical and thermal stability, making it an intriguing candidate material for use in aerospace applications under unique environmental circumstances.

## 2. Materials and Methods

### 2.1. Chemicals

The petroleum-based epoxy resin E51 was from Nantong Xingchen Synthetic Material Co., Ltd. (Jiangsu, China); the TBD, and SA came from Tianjin HEOWNS Biochemical Technology Co., Ltd. (Tianjin, China); and the PA came from Shanghai Aladdin Biochemical Technology Co., Ltd. (Shanghai, China). All chemicals used were of analytical grade.

### 2.2. Synthesis

At ambient temperature, a total of 4 g of epoxy resin E51 and a varying amount of SA (ranging from 0.21 to 1.23 g) were carefully measured and placed into a container. The container, which had a metal bowl coated with polytetrafluoroethylene on the inner wall, was then positioned in a magnetic stirring heating sleeve and heated to a constant temperature of 135 °C for a duration of 5 min. This heating process facilitated the melting of the epoxy resin E51 and the SA. Subsequently, the heating was discontinued, and a quantity of PA (ranging from 1.21 to 2.72 g) was added to the mixture. Continuous agitation was maintained throughout the cooling process to ensure that the PA melted and formed a eutectic with the E51 and SA. Once the eutectic temperature reached 125 °C, 0.28 g of TBD was introduced into the container. After 30 s of vigorous stirring, the container was promptly removed to prevent the excessive polymerization of the prepolymer. The resulting prepolymer mass of 2 to 3 g was then weighed and placed on a die specifically designed for plate pressing. The EV material was obtained by subjecting the prepolymer to hot pressing at a temperature of 180 °C and a pressure of 4 MPa for a duration of 4 h using a hot press [3]. The quantities of the raw materials used for each EV are given in Table 1.

### 2.3. Characterization

Differential scanning calorimetry (DSC) was performed on a DSC instrument (Q2000, TA Instruments, Newark, DE, USA), utilizing a standard aluminum crucible. The sample weight was carefully controlled within the 6–10 mg range. The temperature range for the DSC test was set from 0 to 200 °C, with a heating rate of 10 °C/min. The entire test was carried out under a nitrogen atmosphere.

Stress–relaxation experiments were conducted on a USA TA Instruments rheometer (AR-G2, TA Instruments, Newark, DE, USA) in torsion geometry with 8 mm diameter samples. An axial force of −0.01 N and a deformation of 1% were applied. The relaxation times were measured for 63% relaxation.

The tensile properties were evaluated using an electronic universal material testing machine (5943, Instron, Boston, MA, USA). Rectangular samples measuring 40 × 10 × 0.5 mm^3^ were cut from the EV material and placed on the machine. The test procedure involved gradually increasing the axial tension from 0 N at a rate of 30 N/min until the sample fractured.

The surface morphology was examined using a scanning electron microscope (SEM, Quattro C, Thermo Fisher Scientific, Waltham, MA, USA).

Thermogravimetric (TG) and differential thermal gravimetric (DTG) analyses were performed using a thermogravimeter (TGA-Q50, TA Instruments, Newark, Delaware, USA). The EV material was subjected to a gradual heating process in a nitrogen atmosphere, reaching a maximum temperature of 740 °C at a heating rate of 10 °C/min.

## 3. Results and Discussion

### 3.1. Proposed Mechanisms of the Curing Reactions

**Figure 2:**
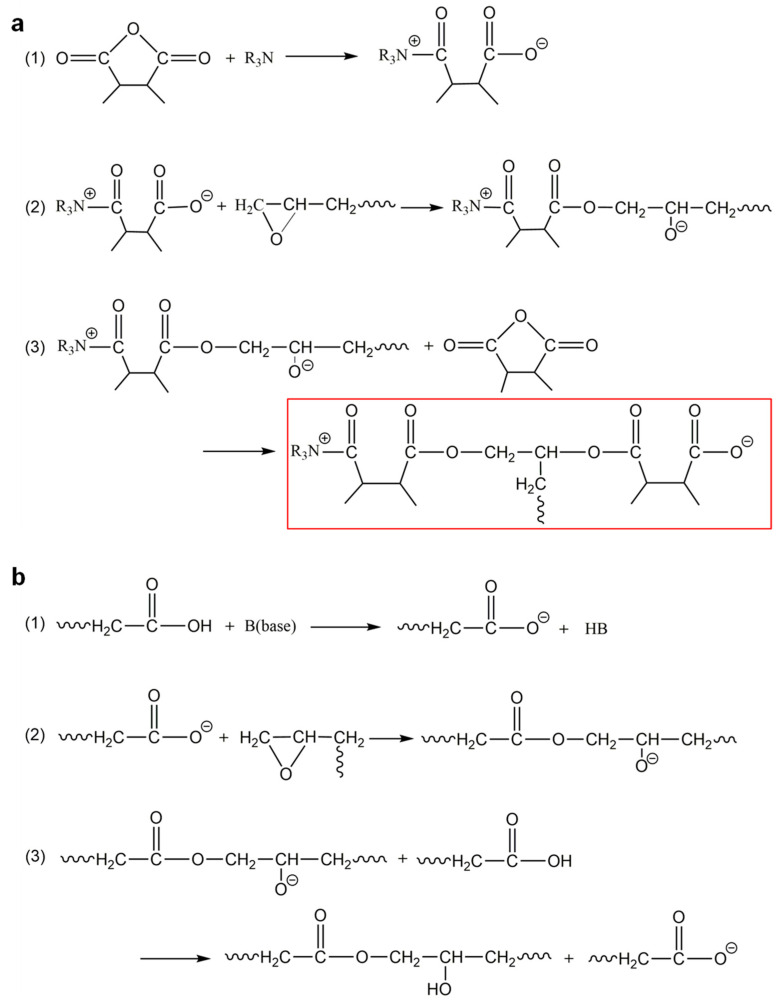
Proposed reaction steps between epoxy and (**a**) anhydrides catalyzed by bases (take R_3_N as the example); (**b**) carboxylic acids catalyzed by bases (abbreviated as B).

The potential mechanisms underlying the reactions between epoxy groups and anhydrides or carboxylic acids, catalyzed by bases, are depicted in Figure 2. Figure 2a illustrates various reaction steps involving the epoxy group and the anhydride, with a base (take R_3_N as the example) as the catalyst. Firstly, the anhydride reacts with R_3_N, resulting in the formation of a carboxylate anion. Subsequently, the carboxylate anion reacts with the epoxy group, causing the opening of the epoxy group and the formation of an alkoxide anion. Finally, the alkoxide anion reacts with another anhydride, leading to the formation of a new carboxylate anion [41]. Similarly, Figure 2b demonstrates the reaction steps between the epoxy group and the carboxylic acid, catalyzed by a base (abbreviated as B). Initially, the carboxylic acid reacts with the B, resulting in the formation of a carboxylate anion. Subsequently, the carboxylate anion reacts with the epoxy group, leading to the opening of the epoxy group and the formation of an alkoxide anion. Finally, a new reaction occurs between the alkoxide anion and another carboxylic acid.

However, upon comparing step 3 in Figure 2a with step 3 in Figure 2b, it becomes evident that the use of anhydrides as a curing agent leads to the formation of carboxylate anions (marked with the red box), which will subsequently react with epoxy groups. This results in the repetition of steps 2 and 3, leading to the creation of new branch structures on the polymer chain. Consequently, the cross-link density of the cured product is increased. Conversely, when carboxylic acids are employed as a curing agent, the reaction with epoxy groups does not yield such branch structures. Therefore, by adjusting the ratio of anhydrides to carboxylic acids in the curing agent, it is possible to modify the cross-link density and, consequently, the *T*_g_ of the resulting EV material.

### 3.2. T_g_s of EV Materials

**Figure 3:**
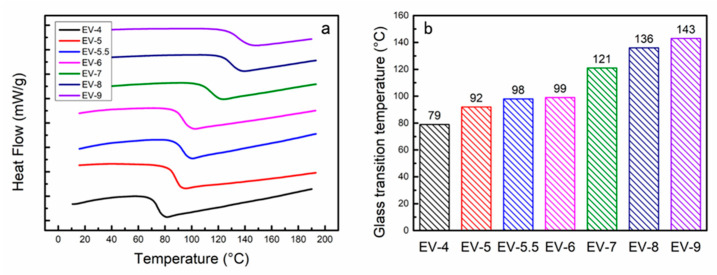
(**a**) DSC curves and (**b**) *T*_g_s of EVs prepared with different ratios of anhydride to carboxylic acid as curing agents.

To investigate the effect of the curing agent on the *T*_g_ of the vitrimer, DSC was performed on the EV materials cured with varying levels of anhydride content in the curing agent. Figure 3a displays the DSC curves for the different EV materials, and Figure 3b illustrates their detailed *T*_g_s. As depicted in Figure 3, the *T*_g_s of the EVs gradually increased from 79 to 143 °C as the proportion of anhydrides in the curing agents increased. This can be attributed to two primary factors. First, the presence of rigid benzene rings in PA leads to significant steric hindrance, thereby raising the energy barrier for molecular chain rotation. Second, in contrast to carboxylic acids, the reaction of anhydride with epoxy groups, facilitated by a base catalyst, enhances the cross-linkage density of cured EV materials. Consequently, when the total molar amount of anhydride and carboxyl groups in the curing agent and the molar amount of epoxy groups in the epoxy oligomer were maintained at a 1:1 ratio, the higher the proportion of PA in the curing agent, the higher the *T*_g_ of the cured EV. The *T*_g_ of the cured EV increased beyond 90 °C when the molar ratio of anhydride to carboxyl groups in the curing agent was 5:5, which met the basic material requirements for low-orbit spaceflight [32].

### 3.3. Rheological Properties of EV Materials

**Figure 4:**
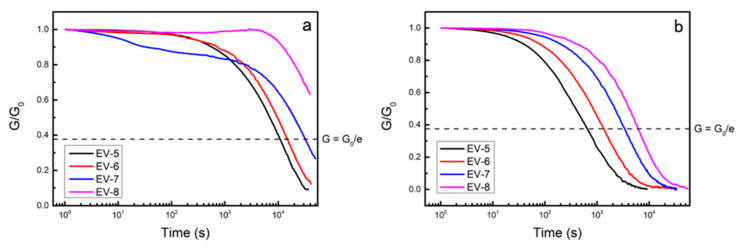
Stress–relaxation curves of several EV materials with *T*_g_ > 90 °C at different temperatures: (**a**) 150 °C, (**b**) 180 °C.

In order to investigate the rheological properties of EV materials at elevated temperatures, shear stress relaxation experiments were conducted on EV materials with *T*_g_s exceeding 90 °C at 150 and 180 °C. The results presented in Figure 4 and Table 2 demonstrate that the majority of EV materials exhibited stress relaxation within the duration of the test (40,000 s) at both temperatures. This suggests that the EV materials were capable of undergoing a TER at higher temperatures, leading to changes in the cross-linked network’s topology. Notably, the relaxation times of EV-5, EV-6, and EV-7 at 180 °C were significantly shorter than those at 150 °C. This observation can be attributed to the accelerated TER rate at elevated temperatures, which expedites the rearrangement process of the cross-linked network.

Furthermore, the relaxation curves at the same test temperature reveal that the higher the content of PA in the curing agent, the longer the relaxation time of the cured EV material. This phenomenon can be attributed to the molecular structure of PA and its reaction characteristics with the epoxy group. In the presence of the base catalyst, the reaction between carboxylic acid and epoxy oligomers efficiently generates two reactants, namely the ester group and the hydroxyl group, facilitating transesterification. Conversely, the reaction between anhydride and epoxy oligomers, facilitated by the base catalyst, only produces ester groups. Consequently, a higher content of anhydride in the curing agent leads to an unfavorable group composition in the curing product for the TER. Additionally, PA possesses a rigid benzene ring structure with significant steric hindrance, which hinders contact between the ester groups and hydroxyl groups in EV materials. Based on the aforementioned findings, it can be concluded that a higher amount of PA in the curing agent hampered the TER of the prepared EV material and slowed down the stress relaxation process. One thing to note here is that the principle of shear rheology testing is based on some basic assumptions; i.e., only when the input or output strain or stress is applied to the sample, and the flow field is a pure shear flow field, the test results are reliable, so different samples often need to adopt their own specific test conditions and techniques to ensure that these basic assumptions are valid [42]. The set of samples (EV-5, EV-6, EV-7, and EV-8) used here to determine the relaxation times were different, but in order to ensure the comparability of the test results, the same test conditions were used, which may affect the accuracy of the absolute values of the relaxation times for the samples. Therefore, the absolute values of the relaxation times here are suggested to be neglected and their relative values are considered. Comprehensively considering the performance requirements of a *T*_g_ above 90 °C and a shorter stress relaxation time, the material EV-5.5 should be able to meet the criteria. Thus, EV-5.5 was primarily evaluated in the follow-up work.

**Figure 5:**
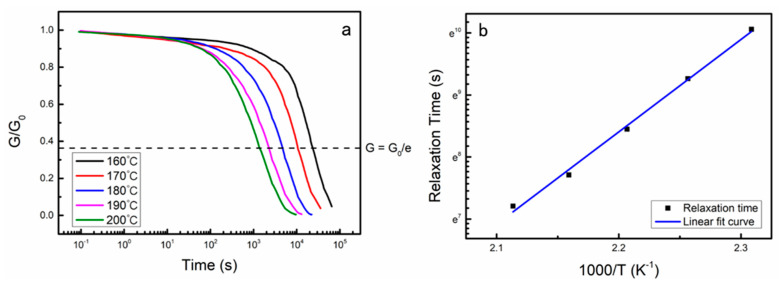
(**a**) Normalized stress–relaxation curves of the EV-5.5 material at different temperatures; (**b**) fitting of the relaxation times to the Arrhenius equation.

Shear stress relaxation experiments were conducted on EV-5.5 at various temperatures, ranging from 160 to 200 °C. The stress relaxation curves of the EV-5.5 material at different temperatures are depicted in Figure 5a, while the corresponding relaxation times are presented in Table 3. The experimental findings indicate that, as the shear stress relaxation test temperature increased from 160 to 200 °C, the relaxation time of the EV-5.5 material gradually decreased from 23,354 s to 1350 s. This observation suggests that the rate of the topological transition of the cross-linked network within the material accelerates with increasing temperature. The test results further indicate that the correlation between the relaxation time (*τ*) and the shear rheological test temperature (*T*) for the EV-5.5 material adheres to the Arrhenius equation, ln*τ* = ln*τ*_0_ + *E_a_*/*RT*. Specifically, the ln*τ* at each temperature exhibits a linear relationship with 1000/*T*, as depicted in Figure 5b. This finding provides further evidence that the EV materials possessed the rheological characteristics that are commonly observed in vitrimer materials at elevated temperatures [30]. By analyzing the slope of the fitted curve and employing the Arrhenius equation, the activation energy (*E_a_*) associated with the relaxation process of the EV-5.5 material was determined to be 123.8 kJ/mol, which was in good agreement with those reported by Leibler and other researchers (69–150 kJ mol^−1^) [35]. Compared with V4 with glycyrrhizic acid as the component curing agent, as reported by Wu et al. [37], EV-5.5 exhibited a higher *T*_g_ and a slower relaxation rate, which was capable of meeting the requirements for our target applications in aerospace.

### 3.4. Mechanical Properties of EV-5.5

**Figure 6:**
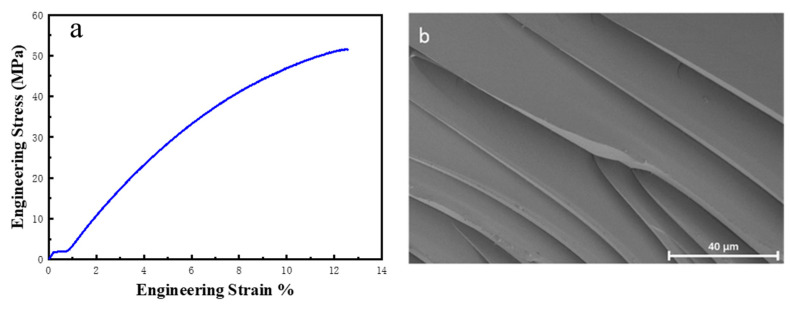
(**a**) Tensile curve of EV-5.5; (**b**) SEM image of the rupture cross-section.

To assess the mechanical properties of the EV material, EV-5.5 was subjected to a tensile test at room temperature. The resulting tensile curve is depicted in Figure 6a. The graph illustrates the linear relationship between stress and strain with increasing applied load. Upon reaching an applied load of 52 ± 6 MPa, the material experienced a rupture, exhibiting a 12.5 ± 1.9% elongation at the rupture and a rupture energy of 3.83 ± 1.02 MJ/m^3^.

The tensile curve does not display any stress peaks or plateaus prior to the material’s rupture, implying the absence of significant yield phenomena; namely, the EV-5.5 material exhibited typical elastomeric behavior throughout the stretching process, with brittle breaking occurring before yielding. This observation is further supported in the SEM image of the rupture’s cross-section, as displayed in Figure 6b. The fracture surface of EV-5.5 exhibited smooth and well-ordered streamer-like lines, indicating the occurrence of stress concentrations during tensile processes and confirming the brittle fracture behavior. Compared with the reported V4 and a few bio-based vitrimers, EV-5.5 showed a significantly better tensile strength [37].

### 3.5. Thermal Stabilities of EV-5.5

**Figure 7:**
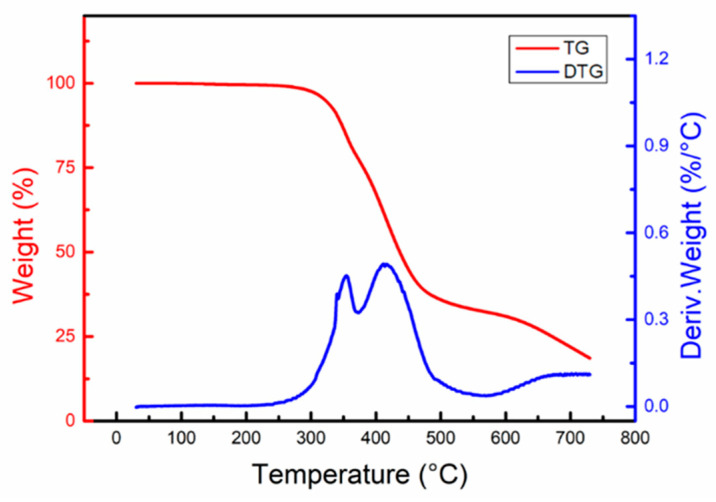
TG curve (red line) and DTG curve (blue line) of the EV-5.5 material.

The thermal stability of EV-5.5 was characterized using TG analysis and the results are presented in Figure 7. The red TG curve shows how the weight of the EV-5.5 sample decreased when it was heated. The initial decomposition temperature of EV-5.5 was determined to be 260 °C. Within the temperature range of 0–260 °C, the sample experienced a weight loss of 0.5%, which is attributed to the volatilization of the TBD catalyst, the unreacted curing agent, and other substances. As the temperature increased, the decomposition rate of EV-5.5 accelerated, with decomposition temperatures of 322, 341, and 437 °C corresponding to weight losses of 5%, 10%, and 50%, respectively. The EV-5.5 material demonstrated good thermal stability, and the temperature at 5% weight loss (*T*_d5_) was substantially higher than 263 °C [37]. By performing differential calculations on the TG curve, the DTG curve (blue line) was obtained, which provides insight into the decomposition rate of the materials at different temperatures. The DTG curve reveals that EV-5.5 decomposed rapidly within the temperature range of 300–500 °C, with the decomposition rates peaking at 354 and 415 °C.

## 4. Conclusions

The EV materials were synthesized using epoxy E51 as the base resin, with PA and SA as the curing agents and TBD as the catalyst. Shear rheology tests revealed that the EV materials exhibited stress relaxation behavior at high temperatures, and the relaxation time was found to be consistent with that of vitrimer materials. DSC and shear rheology tests demonstrated that the *T*_g_ of the cured EV material increased, and the fluidity decreased with higher anhydride contents in the curing agent. This can be attributed to the larger steric hindrance of the molecular structure of PA, which leads to an increased cross-linking density in the cured product when reacted with epoxy under TBD catalysis. Tensile tests revealed that the EV-5.5 sample displayed elastomeric properties, with brittle breaking occurring before yielding, which exhibited a tensile strength of 52 MPa, an elongation at fracture of 12.5%, and a fracture energy of 3.83 MJ/m^3^. The initial decomposition temperature of EV-5.5 was 260 °C, according to the TG analysis data. Two decomposition peaks appeared as the temperature rose, at 354 and 415 °C, respectively, and the decomposition temperatures of 5%, 10%, and 50% were 322 °C, 341 °C, and 437 °C, respectively. This study, aiming to prepare EV materials with enhanced and tunable *T*_g_s, opens up new opportunities for thermosets’ practical applications.

## Figures and Tables

**Table 1:** Feed compositions of each EV (the relative relationship between the moles of groups or molecules and the mole number of the epoxy group is set to 10).

Label	Epoxy Group (E51)	Anhydride Group (PA)	Carboxyl Group (SA)	TBD
EV-4	10	4	6	1
EV-5	10	5	5	1
EV-5.5	10	5.5	4.5	1
EV-6	10	6	4	1
EV-7	10	7	3	1
EV-8	10	8	2	1
EV-9	10	9	1	1

**Table 2:** Relaxation time of each EV (*T*_g_ > 90 °C) in the shear rheological test at different temperatures.

Test Temperature (°C)	Relaxation Time (s)			
	EV-5	EV-6	EV-7	EV-8
150	10,872	15,170	32,800	>40,000
180	660	1334	3460	6170

**Table 3:** Relaxation times for the EV-5.5 material at various temperatures.

Test temperature (°C)	160	170	180	190	200
Relaxation time, *τ* (s)	23,354	10,536	4664	2231	1350

## Data Availability

The authors confirm that the data supporting the findings of this study are available within the article.
